# Efficacy and safety of flumatinib in adults with Ph-positive ALL: a prospective observational study

**DOI:** 10.3389/fonc.2025.1721146

**Published:** 2025-12-09

**Authors:** Jianmei Xu, Jing Wang, Songying Zhao, Huimei Guo, Jiangbo Zhang, Jia Liu, Luoming Hua, Hua Xue

**Affiliations:** Department of Hematology, Affiliated Hospital of Hebei University, Baoding, China

**Keywords:** acute lymphoblastic leukemia, Philadelphia chromosome, flumatinib, minimal residual disease, treatment outcome

## Abstract

**Objective:**

To evaluate the efficacy and safety of flumatinib, a second-generation tyrosine kinase inhibitor (TKI), combined with chemotherapy in the treatment of Philadelphia chromosome-positive acute lymphoblastic leukemia (Ph+ ALL), and to analyze factors influencing prognosis.

**Methods:**

This prospective, single-center, observational study included 15 newly diagnosed adult Ph+ ALL patients admitted between January 2022 and December 2024. All patients received a flumatinib-based combination chemotherapy regimen (600 mg once daily). The primary outcomes included complete remission (CR) rate, negativity rates for fusion gene and flow cytometry-based minimal residual disease (MRD), progression-free survival (PFS), overall survival (OS), and adverse events (AEs).

**Results:**

Among the 15 patients, 14 achieved hematological complete remission (93.3%). At the end of induction therapy, the fusion gene and flow-MRD negativity rates were 60.0% (9/15) and 73.3% (11/15), respectively. By 3 months of treatment, the cumulative negativity rates increased to 80.0% (12/15) and 86.7% (13/15), respectively. With a median follow-up of 26 months, the median PFS and OS were 11 months (range: 1–60) and 24 months (range: 6–60), respectively. Subgroup analysis revealed that two patients with chronic myeloid leukemia in lymphoid blast phase (CML-LBP) had extremely poor outcomes, with median PFS and OS of only 3 months and 7.5 months, respectively. The most common grade 3–4 adverse events were hematological toxicities (53.3%), followed by infections and liver function abnormalities. All AEs were manageable with supportive care, and no treatment-related deaths occurred.

**Conclusion:**

Flumatinib combined with chemotherapy induced high remission rates and deep molecular responses in newly diagnosed Ph+ ALL patients, with a favorable safety profile. However, patients with CML-LBP or high-risk Ph+ ALL had poor treatment responses and outcomes, indicating the need for more aggressive intervention strategies in this high-risk population.

## 1 Introduction

Philadelphia chromosome-positive acute lymphoblastic leukemia (Ph+ ALL), resulting from the t(9;22)(q34;q11) translocation and formation of the BCR-ABL fusion gene, accounts for 20%–40% of adult ALL cases (1). It is characterized by aggressive progression, poor response to conventional chemotherapy, and high relapse rates, with a historical 5-year overall survival (OS) rate of less than 10% with chemotherapy alone (2). The introduction of tyrosine kinase inhibitors (TKIs) has markedly improved outcomes in Ph+ ALL. Second-generation TKIs such as dasatinib and nilotinib have further increased complete remission (CR) and molecular response rates, leading to 5-year OS rates of 40%–70% (3–5).

Flumatinib is a novel second-generation TKI developed through structural optimization of imatinib—including pyridine ring substitution and the introduction of a trifluoromethyl group—resulting in enhanced binding affinity and selectivity for BCR-ABL kinase (6). Preclinical studies have demonstrated its potent inhibitory activity against various ABL kinase mutations, such as V299L and F317L (7). Although flumatinib has shown superior efficacy and safety compared to imatinib in chronic myeloid leukemia (CML), clinical data regarding its use in Ph+ ALL remain limited.

To date, studies of flumatinib in Ph+ ALL have primarily been small retrospective analyses. Liu et al. reported that flumatinib combined with chemotherapy led to higher rates of complete molecular response (CMR) and minimal residual disease (MRD) negativity within three months, along with a favorable adverse event profile (8). Further work by Wang et al. demonstrated that flumatinib effectively penetrates the blood-brain barrier and exerts potent pro-apoptotic and anti-proliferative effects on Ph+ ALL cell lines such as SUP-B15 (9).

Although preliminary evidence suggests that flumatinib holds promise for the treatment of Ph+ ALL, more real-world clinical data are needed to validate its efficacy and safety. This study aims to provide the valuable evidence on the efficacy and safety of flumatinib-based regimens, with a particular focus on molecular responses and outcomes in high-risk subgroups.

## 2 Materials and methods

### 2.1 Study design and patients

This prospective, single-center, observational study was conducted at the Affiliated Hospital of Hebei University. All enrolled patients were of Chinese ethnicity. The study protocol was approved by the hospital’s Ethics Committee, and all participants provided written informed consent. A total of 15 newly diagnosed adult Ph+ ALL patients were enrolled between January 1, 2022, and December 30, 2024.

### 2.2 Inclusion criteria

1. Age ≥ 18 years;
2. Diagnosis of Ph+ ALL confirmed by cytomorphology, immunophenotyping, cytogenetics (presence of t(9;22)(q34;q11), and molecular testing (BCR-ABL fusion gene);
3. Eligible for and willing to receive flumatinib-based combination chemotherapy;
4. ECOG performance status ≤ 2;
5. Expected survival ≥ 3 months;
6. Provision of signed informed consent.

### 2.3 Exclusion criteria

1. Pregnancy or lactation;
2. Severe uncontrolled cardiac, hepatic, or renal dysfunction;
3. Concurrent active malignancy;
4. Prior TKI or chemotherapy for ALL;
5. Allergy to flumatinib or its excipients;
6. Any other condition deemed unsuitable for study participation by the investigators.

### 2.4 Treatment regimens

All enrolled patients received flumatinib-based (0.6 g, orally, once daily) combination chemotherapy strictly according to the treatment regimens. The specific regimen was tailored based on the patient’s age, performance status, and the presence of significant comorbidities. Patients scheduled for hematopoietic stem cell transplantation were excluded from this study due to economic reasons.

1. For patients aged <55 years or without severe comorbidities:

Induction therapy: The VDCP regimen (Vincristine + Daunorubicin/Idarubicin + Cyclophosphamide + Prednisone) combined with flumatinib (as soon as possible after diagnosis) was administered.

Consolidation and intensification therapy: Sequential therapy included the CAM regimen (Cyclophosphamide + Cytarabine + Mercaptopurine), high-dose methotrexate (HD-MTX), and delayed intensification (VDCD regimen). Flumatinib was administered continuously during all non-myelosuppressive phases of therapy.

Maintenance therapy: Patients who completed the subsequent intensification therapy entered the maintenance phase, which consisted of alternating monthly cycles of flumatinib (administered for 2 weeks per month) and oral 6-MP + MTX. Maintenance therapy was continued for up to 3 years after achieving complete remission (CR).

2. For patients aged ≥55 years, or those with a poor Frail score, or significant comorbidities:

The treatment regimen was appropriately modified to balance efficacy and safety. Modifications included: reduced cyclophosphamide dose (750 mg/m²) during induction, reduced number of days for daunorubicin administration, reduced dose and duration of cytarabine, and shortened HD-MTX infusion time (to 12 hours). Flumatinib was also administered continuously throughout.

3.Central nervous system leukemia (CNSL) prophylaxis:

All patients received triple intrathecal therapy (Methotrexate + Cytarabine + Dexamethasone) for at least 12–16 sessions.

4.Supportive care:

Standard supportive care, including blood component transfusion, hematopoietic growth factors, prophylactic anti-infective agents, and hepatoprotective drugs, was provided as clinically indicated.

5. Observation indicators and evaluation criteria.

Efficacy evaluation: Treatment response was assessed on day 14 and 28 (end of induction) of induction therapy, before each consolidation/intensification cycle, and every 3 months during maintenance therapy. Definitions for Complete Remission (CR), Complete Molecular Response (CMR), and Major Molecular Response (MMR) were based on the NCCN guideline criteria.

Minimal residual disease (MRD) monitoring:

Multiparameter flow cytometry (MFC) and digital PCR (ddPCR) were used to quantitatively detect BCR-ABL fusion gene levels. MRD was monitored during induction therapy, before each consolidation/intensification cycle, and every 3 months during maintenance therapy.

MRD negativity by flow cytometry was defined as < 10^-4^ leukemic cells. MMR was defined as BCR-ABL^(IS) ≤ 0.01%, and CMR was defined as undetectable BCR-ABL transcripts.

Safety evaluation: All adverse events (AEs) occurring during treatment were recorded in detail and graded according to the National Cancer Institute’s Common Terminology Criteria for Adverse Events (NCI CTCAE version 5.0). Special attention was paid to flumatinib-related adverse reactions, such as hematological toxicity, gastrointestinal events, and abnormal liver function.

Survival follow-up: Follow-up was conducted via outpatient reviews and telephone interviews to record Overall Survival (OS) and Progression-Free Survival (PFS). The follow-up cutoff date was June 30, 2025.

6. Statistical methods.

Statistical analyses were performed using SPSS software (version 26.0). Continuous data are presented as mean ± standard deviation or median (range), while categorical data are expressed as number (percentage). A *P*-value < 0.05 was considered statistically significant.

## 3 Results

### 3.1 Patient characteristics

A total of 15 newly diagnosed Ph+ ALL patients were included in this study, comprising 11 females (73.3%) and 4 males (26.7%), with a median age of 55 years (range: 21–67). All patients received induction therapy with flumatinib (600mg/day) combined with a VDCP-based regimen. Baseline clinical characteristics are summarized in **Table 1**.

**Table 1:** The baseline characteristics of the patients.

Characteristics	N/%
Sex	
Male	4 (26.7%)
Female	11 (73.3%)
Age	55 (21–67)
ECOG	
0–1	9 (60%)
2	6 (40%)
WBC	
WBC>30*10^9/L	8(53.3%)
WBC ≤ 30*10^9/L	7(46.7%)
Fusion gene type	
P190	12 (80%)
P210	3 (20%)
Complex karyotype	4 (26.7%)
NGS	
IKZF1	1 (6.7%)
TP53	1 (6.7%)

ECOG, Eastern Cooperative Oncology Group; WBC, White blood cell; NGS, Next-generation sequencing.

### 3.2 Treatment response and MRD negativity

**Figure 1:**
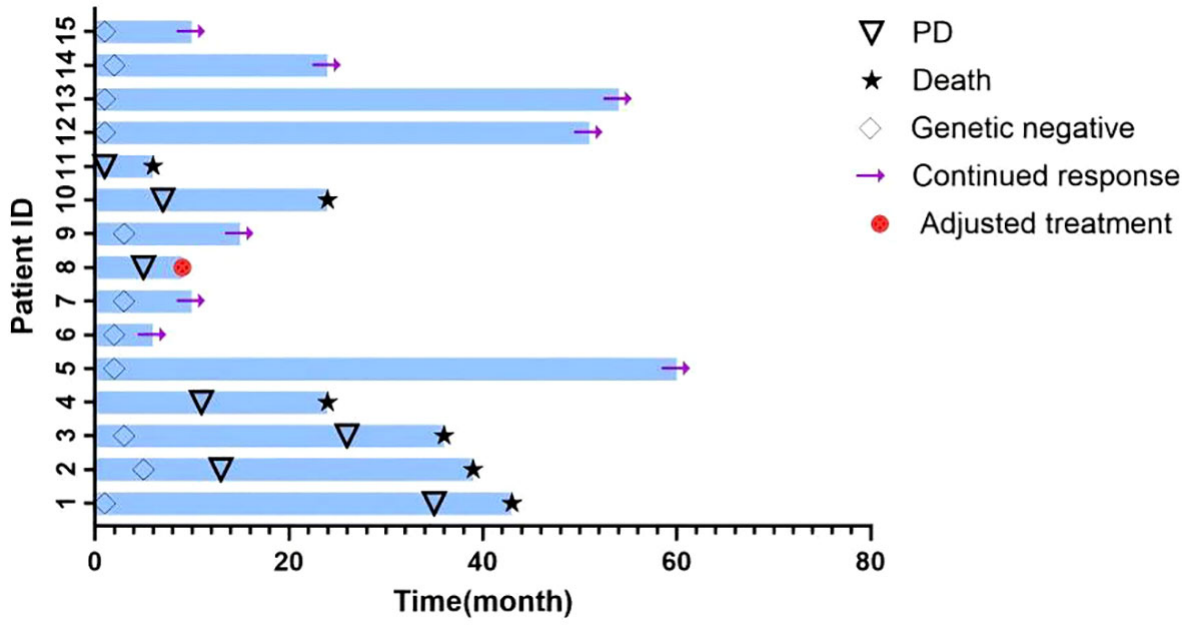
The clinical course and outcomes of 15 Ph+ ALL patients treated with flumatinib-based chemotherapy. The swimmer’s plot illustrates individual patient timelines from initiation of therapy. Each horizontal bar represents one patient. Symbols indicate key clinical events. Genetic negativity: BCR-ABL fusion gene undetectable. PD: disease progression. Adjusted treatment: switch to alternative TKI or salvage therapy.

Fourteen of the 15 patients (93.3%) achieved hematological complete remission (CR) by the end of induction therapy. One patient (Case 11) with CML-LBP exhibited primary resistance and did not achieve CR. The conditions of all 15 patients are shown in **Figure 1**.

#### 3.2.1 Fusion gene negativity

**Figure 2:**
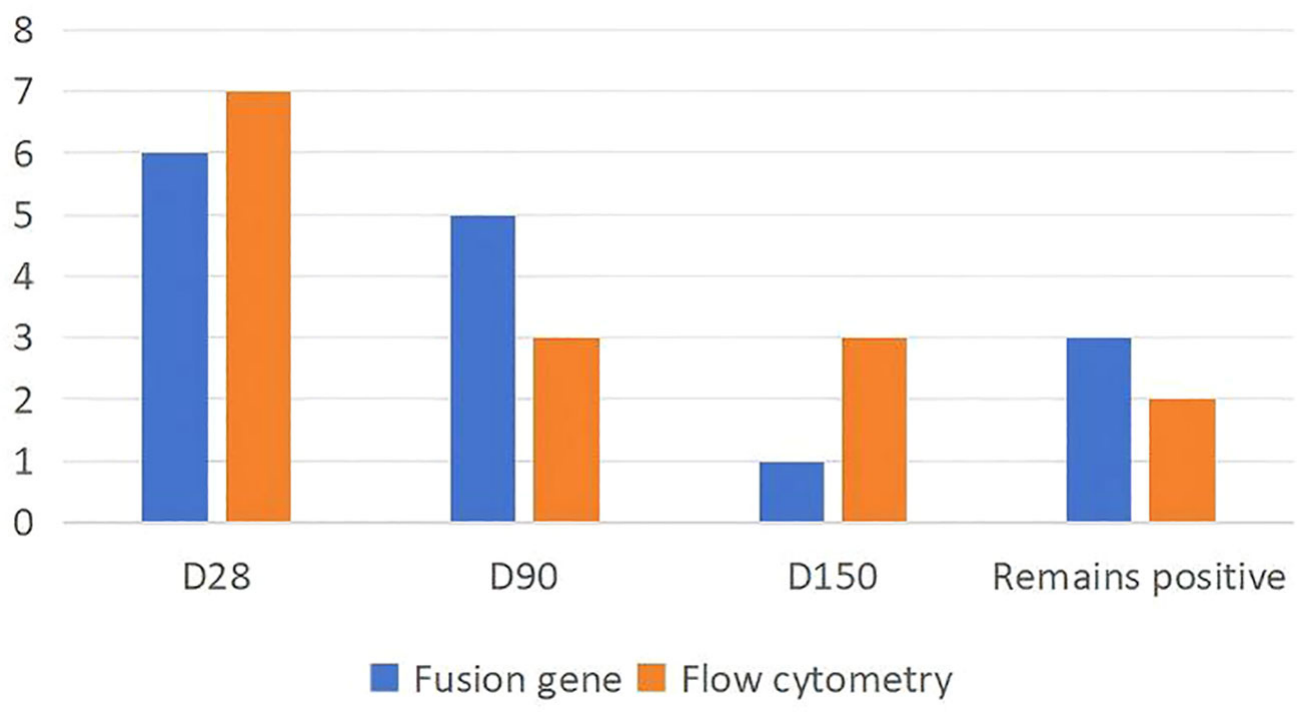
The dynamics of MRD negativity during treatment. Stacked bar chart showing the proportion of patients achieving MRD negativity by fusion gene (ddPCR) and flow cytometry at key timepoints: end of induction (D28), 3 months (D90), and 5 months (D150). The “Remains positive” category indicates patients who did not achieve MRD negativity during follow-up.

At the end of induction (day 28), the fusion gene negativity rate was 60.0% (9/15). The cumulative negativity rate increased to 80.0% (12/15) within 3 months. Three patients (20.0%) did not achieve fusion gene negativity, including one case with primary resistance. The median time to fusion gene negativity was 2 cycles. For specific details, please refer to **Table 2**, **Figure 2**.

**Table 2:** The situation of MRD turning negative after the induction treatment.

Time	Reversion rate of integrated gene (%)	Flow cytometry-based MRD negativity rate (%)
At the end of the induction therapy	9/15 (60.0%)	11/15 (73.3%)
Within three months	12/15 (80.0%)	13/15 (86.7%)
Always remains positive (not negative)	3/15 (20.0%)	2/15 (13.3%)

#### 3.2.2 Flow cytometric MRD negativity

At the end of induction therapy, the rate of MRD negativity by flow cytometry was 73.3% (11/15). The cumulative MRD negativity rate within 3 months reached 86.7% (13/15). Two patients (13.3%) remained MRD-positive throughout. The median time to achieving flow cytometric MRD negativity was at the completion of induction therapy. For specific details, please refer to **Table 2**, **Figure 1**.

### 3.2.3 Subgroup analysis

Patients presenting with hyperleukocytosis (white blood cell count > 30 × 10^9^/L) at diagnosis had significantly higher rates of disease progression and mortality (87.5% vs. 28.6%) compared to patients with normal initial WBC. Next-generation sequencing (NGS) identified high-risk genetic alterations (e.g., IKZF1, TP53, EVI1/ABL fusion) in 5 patients. With the exception of one long-term survivor (Case 13, with EZH2 and NOTCH1 mutations), all patients carrying these high-risk mutations experienced early disease progression or death, suggesting that TP53 mutations and EVI1 rearrangements may serve as poor prognostic predictors in this patient population.

Furthermore, two patients in the cohort were diagnosed with chronic myeloid leukemia in lymphoid blast phase (CML-LBP) (Cases 8 and 11), which is a distinct disease entity from *de novo* Ph+ ALL but was included in our cohort of “newly-diagnosed” Ph+ ALL patients due to its similar initial presentation. This subgroup exhibited more aggressive disease features and inferior treatment responses.

Case 8: Although hematological complete remission (CR) was achieved after induction therapy, the BCR-ABL fusion gene remained persistently detectable. The patient experienced disease progression 5 months later, necessitating a change in therapy.

Case 11: This patient exhibited primary resistance to treatment. Following induction therapy, the BCR-ABL transcript level (P210 isoform) remained high at 105.56%, flow cytometric MRD was persistently positive, and CR was not achieved. Disease progression occurred within one month, and the patient died at 6 months.

### 3.3 Survival outcomes and relapse

With a median follow-up of 26 months, the median progression-free survival (PFS) was 11 months and median overall survival (OS) was 24 months. By the last follow-up, 8 patients were alive and 7 had died. A total of 8 patients experienced disease progression or relapse, 5 of whom (Cases 4, 8, 10, 11, 12) relapsed early (within 12 months; median PFS 7.5 months). Causes of relapse included CNS involvement (n = 1), disease progression (n = 3), self-discontinuation of maintenance therapy (n = 1), and failure to achieve remission (n = 1). The primary cause of death was disease progression or relapse.

**3.**4 Adverse events.

**Figure 3:**
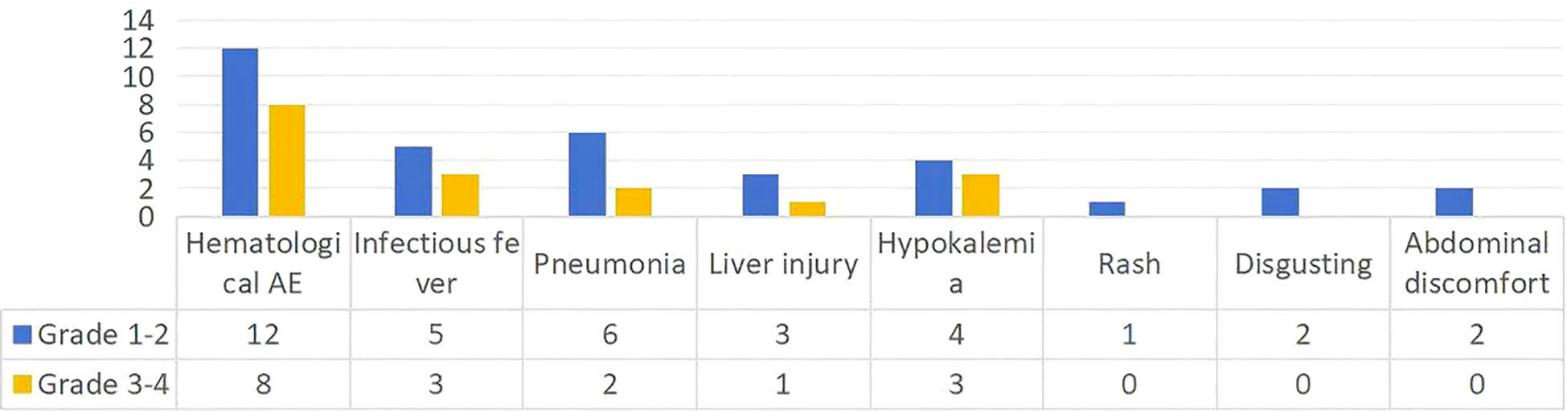
The spectrum and severity of adverse events in 15 Ph+ ALL patients. The bar graph depicts the frequency and grade (1–2 vs. 3–4) of adverse events (AEs) according to NCI CTCAE v5.0. Hematological AEs were the most common severe toxicities, followed by infectious and hepatic events. No grade 5 AEs were observed.

The most common adverse events were hematologic toxicities (grade 3–4, n = 8, 53.3%), followed by infections and liver function abnormalities. Most AEs were manageable with supportive care, and no treatment-related deaths occurred. During flumatinib monotherapy, two patients experienced abdominal discomfort and nausea, with no other significant AEs reported. For specific details, please refer to **Figure 3**.

## 4 Discussion

As a novel second-generation TKI, flumatinib has demonstrated efficacy in chronic myeloid leukemia (CML), yet its clinical profile in Ph^+^ ALL remains less established (9, 10). This prospective study of 15 Ph^+^ ALL patients treated with flumatinib plus chemotherapy provides further evidence supporting its efficacy and safety in this setting.

Our results showed that 93.3% patients achieved hematological CR, with fusion gene negativity rates of 60.0% and flow-MRD negativity of 73.3% at the end of induction. These rates increased to 80.0% and 86.7%, respectively, within three months. After a median follow-up of 26 months, median OS and PFS were 24 and 11 months, respectively. Toxicities were primarily hematological and generally manageable. These findings reinforce the potential of flumatinib as an effective and well-tolerated frontline therapy for Ph^+^ ALL.

Our data are consistent with previous reports. Wang et al. observed MRD negativity in 82.6% of 29 Ph^+^ ALL patients at 1 month, increasing to 95.6% at 3 months (9). Similarly, Zhang et al. reported a CR rate of 96.9% after induction and a cumulative major molecular response (MMR) rate of 56.6% at 3 months (10). Although our sample size is limited, the flow-MRD negativity rate at the end of induction (73.3%) aligns with these studies, supporting the role of flumatinib in inducing rapid and deep molecular responses—likely attributable to its enhanced BCR-ABL1 inhibitory potency.

Although our study did not directly measure flumatinib concentrations in cerebrospinal fluid (CSF), previous work by Wang et al. reported detectable flumatinib levels in CSF (0.67 ± 0.61 ng/mL), with a penetration rate of 3.01%, significantly higher than that of dasatinib (<1 ng/mL) (9). This property may contribute to effective central nervous system (CNS) prophylaxis (11). Consistent with this, only one patient in our cohort experienced CNS relapse.

In line with established prognostic factors in ALL, we found that initial hyperleukocytosis (WBC > 30×10^9^/L) was strongly associated with inferior PFS and OS (12). Moreover, integrated NGS profiling identified high-risk genetic alterations—such as TP53 mutations (13) and EVI1 rearrangements (14)—in a subset of patients who experienced early treatment failure. This underscores the critical importance of comprehensive genetic profiling at diagnosis for improved risk stratification. Patients harboring these high-risk features may represent a distinct subgroup that derives limited benefit from flumatinib-based chemotherapy alone and should be considered for more intensive or novel strategies—such as allogeneic hematopoietic stem cell transplantation (HSCT) or third-generation TKIs—early during treatment (15, 16).

Notably, two patients in our cohort had CML in lymphoid blast phase (CML-LBP), both exhibiting aggressive clinical courses and poor responses. One achieved hematologic CR but remained fusion gene-positive, progressing at 5 months; the other exhibited primary resistance and died within 6 months. This subgroup had significantly worse outcomes than *de novo* Ph^+^ ALL patients, echoing findings from large studies which highlight the dismal prognosis of CML-LBP and the prevalence of high-risk mutations such as T315I and Y253H that drive resistance (17). These results suggest that third-generation TKIs (e.g., olverembatinib (18) or ponatinib (19) and/or HSCT should be considered earlier in this population.

Regarding safety, the most common AEs were hematologic toxicities (53.3% grade 3–4), followed by infections and hepatic dysfunction. Most were manageable with supportive care, and no treatment-related deaths occurred. Compared with dasatinib, flumatinib appears associated with a lower incidence of non-hematologic AEs—such as pleural effusion, pancreatitis, and diarrhea—suggesting a more favorable tolerability profile.

Regarding safety, the most common AEs were hematologic toxicities (53.3% grade 3-4), followed by infections and hepatic dysfunction, which is consistent with the known safety profile of flumatinib (7). Most were manageable with supportive care, and no treatment-related deaths occurred. Compared with dasatinib, flumatinib appears associated with a lower incidence of non-hematologic AEs—such as pleural effusion, pancreatitis, and diarrhea (toxicities commonly associated with dasatinib (2)—as directly evidenced in a comparative study (8). This more favorable tolerability profile may be attributed to flumatinib’s distinct kinase inhibition spectrum, particularly its lack of inhibition against off-targets like c-Src (6).

Despite limitations including a small sample size and single-center design, which may restrict the generalizability of our findings and introduce potential selection bias, our findings are consistent with emerging evidence and support the clinical utility of flumatinib in Ph^+^ ALL. Larger multicenter randomized trials are warranted to directly compare flumatinib with other second-generation TKIs (e.g., dasatinib, nilotinib) and to optimize treatment strategies for high-risk subgroups, including those with CML-LBP, IKZF1 deletion, or TP53 mutation.

## Data Availability

The original contributions presented in the study are included in the article/supplementary material. Further inquiries can be directed to the corresponding author.
